# The C-terminal residue of phage *Vp*16 PDF, the smallest peptide deformylase, acts as an offset element locking the active conformation

**DOI:** 10.1038/s41598-017-11329-3

**Published:** 2017-09-08

**Authors:** Renata Grzela, Julien Nusbaum, Sonia Fieulaine, Francesco Lavecchia, Willy V. Bienvenut, Cyril Dian, Thierry Meinnel, Carmela Giglione

**Affiliations:** 1 Institute for Integrative Biology of the Cell (I2BC), CEA, CNRS, Univ. Paris-Sud, Université Paris-Saclay, 91198 Gif-sur-Yvette Cedex, Paris, France; 2 Present Address: Centre of New Technologies, University of Warsaw, S. Banacha 2c, 02-097 Warsaw, Poland

## Abstract

Prokaryotic proteins must be deformylated before the removal of their first methionine. Peptide deformylase (PDF) is indispensable and guarantees this mechanism. Recent metagenomics studies revealed new idiosyncratic PDF forms as the most abundant family of viral sequences. Little is known regarding these viral PDFs, including the capacity of the corresponding encoded proteins to ensure deformylase activity. We provide here the first evidence that viral PDFs, including the shortest PDF identified to date, *Vp*16 PDF, display deformylase activity *in vivo*, despite the absence of the key ribosome-interacting C-terminal region. Moreover, characterization of phage *Vp*16 PDF underscores unexpected structural and molecular features with the C-terminal Isoleucine residue significantly contributing to deformylase activity both *in vitro* and *in vivo*. This residue fully compensates for the absence of the usual long C-domain. Taken together, these data elucidate an unexpected mechanism of enzyme natural evolution and adaptation within viral sequences.

## Introduction

Peptide deformylases (PDFs) are enzymes involved in a pervasive and essential process called N-terminal methionine excision (NME). During NME, PDFs catalyze the removal of the formyl moiety from initiator methionine, found at the beginning of all prokaryotic translation products. Deformylation involves 95% of the proteins of a bacterial proteome^1^.

PDFs belong to the HEXXH-containing metalloprotease super-family, with high conservation of only 3 sets of residues, which build the active site of the enzyme (motif I, GΦGΦΑAXQ, motif II, EGCXS and motif III, HEΦDHXXG, where Φ is a hydrophobic aliphatic amino acid and X is any amino acid; for a review, see ref. 2). Cys from motif II and the two His of motif III, together with a water molecule, coordinate a metal ion involved in catalysis^3–5^. For decades, PDFs were surmised to be present only in bacteria. However, the surprising discovery of plant PDFs allowed to reveal the presence of these enzymes also in other higher eukaryotes^6^. Three Types (1, 2 and 3) could be progressively defined according to their overall levels of similarities (for review, see refs 2, 7 and 8). In this early classification of PDFs, Type 1 comprised two subgroups, Types 1B and 1A. Type 1B PDF was exemplified by the *E*. *coli* enzyme, showing a globular core and a C-domain involving a 3_10_-helix (referred as η3), common to all known PDF structures, followed by an α-helix (α3)^9, 10^. Interestingly, the α3 helix of *E*. *coli* PDF was shown to be crucial for activity *in vivo* by allowing tight interaction with the ribosome next to the exit tunnel^11^. In addition, significant reduction of the activity *in vitro* was noticed when further trimming the *E*. *coli* PDF C-domain beyond the η3 3_10_-helix made of the 4 residues FMDY, which folding correctly orientates the α3 helix back towards the N-terminal domain^12^. Gram-negative bacteria PDFs all belonged to the Type 1B subgroup. Type 1B PDFs were also found in the organelles of eukaryotes where protein synthesis occurs. Type 1A PDFs, only present in mitochondria, feature a long insertion that partially tethers the active site entrance, contributing to a reduction in the size of the ligand binding pocket, and a C-domain folding in the opposite orientation compared to Type 1B PDFs^13, 14^. Type 2 PDFs were represented in this early classification by *Bacillus stearothermophilus* PDF and also included PDFs from Mycoplasma and Gram-positive bacteria. However, when two PDFs occurred in some Gram-positive bacteria, the second was often a Type 1 PDF. Type 2 PDFs were characterized by several insertions and a completely different fold of the C-domain compared to other PDFs^7^. Finally, Type 3 PDFs clustered the mostly weakly active or inactive PDFs, found in Archaea and in trypanosomatid mitochondria, as they displayed substitutions in one or several crucial amino acids of the conserved motifs^6^. The C-termini of Type 3 PDFs, for which no structure is known, displayed no conservation in length.

Recent high-throughput screening, sequencing and annotation of thousands of genomes have strongly contributed to revolutionizing our perception of the distribution of PDFs among kingdoms, revealing putative PDFs in almost all organisms, including viruses. In particular, the pioneer marine metagenomics study of viruses within oceanic microbial samples retrieved unpredicted modified PDF genes as the most abundant family in most of these phage genomes^15^. Sequence comparisons with other known PDFs reveal that viral PDFs are devoid of the key ribosome-interacting C-terminal region. Despite this initial report and the following study uncovering other phage PDFs, no further characterization and functional studies of these intriguing PDFs have been described thus far, particularly *in vivo*. However, the first sequence alignments of these phage PDFs showing perfect conservation of the motifs of the catalytic residues^15^, together with the recent characterization of *Synechococcus* phage S-SSM7 PDF^16^, prompted the suggestion that all discovered putative phage PDFs might be active enzymes.

To support this trend and enlarge our insufficient knowledge on viral PDFs, we set out to definitively check the deformylase activity of a number of marine viral PDFs, including *Vp*16 PDF, which is derived from bacteriophages originally isolated from *Vibrio parahaemolyticus* strain 16 (*Vp*16)^17^. We provide the first evidence that some viral PDFs, including the shortest PDF identified to date, *Vp*16 PDF, display deformylase activity *in vivo*. Large scale N-terminomics characterization reveals that *Vp*16 PDF has substrate specificity similar to that of other bacterial PDFs. Surprisingly, biochemical and structural data, combined with site-directed mutagenesis analyses, showed that *Vp*16 PDF significantly differs from previously characterized PDFs in terms of their properties, which can be related to its few infrequent peculiarities. This uniqueness, not involving the predictable active site, underscored unexpected structural and molecular characteristics of the last rare residue Ile_137_, which significantly contributes to deformylase activity *in vitro* and *in vivo*, fully compensating for the absence of the otherwise indispensable C-terminal domain.

## Results

### An updated, accurate phylogenetic tree recapitulating the large diversity of PDFs

**Figure 1:**
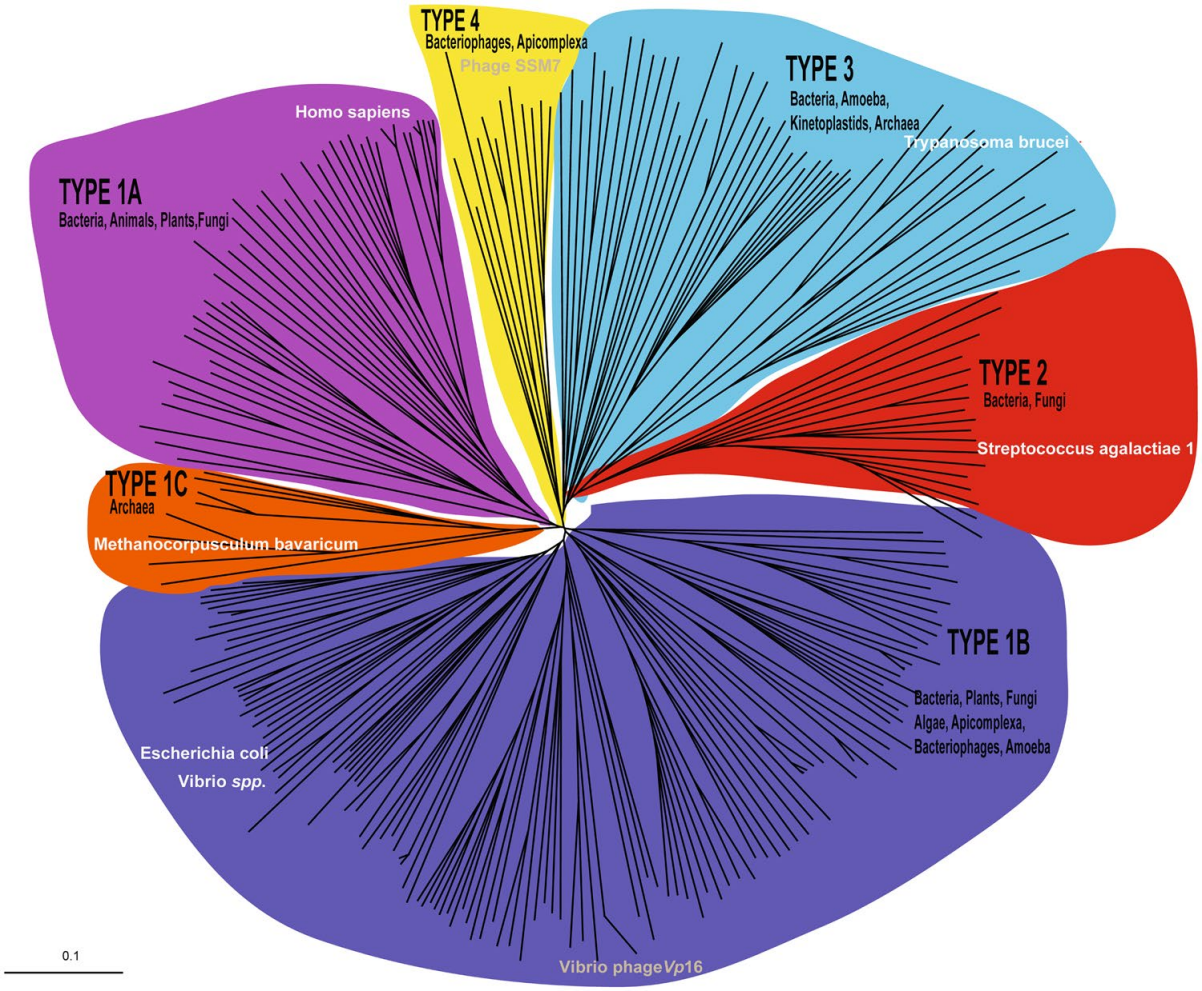
A robust phylogenetic tree of PDFs reveals a new classification for these enzymes. 262 sequences were selected to represent PDF sequence diversity among 20,609 entries. The sequences were aligned with Clustal X^33^ and the bootstrap tree was constructed with PHYLIP. The random number generator seed was 111 and the number of bootstrap trials was 1,000. The rooted phylogenic tree was constructed with N-J Tree and Drawn with TreeView1.66^34^. Internal values labelled on each node record the stability of the branch over the 1,000 bootstrap replicates. Four main PDF Types (PDF1, PDF2, PDF3 and PDF4) and 3 PDF1 subtypes (PDF1A, PDF1B, PDF1C) are clustered and shown in color.

The two first closely related viral deformylase sequences were identified in 2003 in both *Vp*16T and *Vp*16C bacteriophages^17^. The initial classification in a phylogenetic tree of these two putative PDFs, together with other identified viral PDFs, clustered *Vp*16T and *Vp*16C PDFs in a subclass of bacterial PDFs (PDF1B classic), also including *E*. *coli* PDF, not embracing the other viral PDFs assembled in other specific subclasses of PDF1B^15^. A different phylogenetic analysis confirmed the particularity of *Vp*16T and *Vp*16C PDFs, as they did not cluster with other phage PDFs^16^. Given the increasing number of discovered PDF sequences, we built a new accurate phylogenetic tree using sequences selected to represent PDF sequence diversity among the 20,609 entries annotated as “peptide deformylase” available in RefSeq. Only 602 sequences originate from organisms different from bacterial sequences, which represent 97% of the available sequences. Nevertheless, strong redundancy due to metagenomics programs and the many clinical isolates of pathogenic bacteria led us to strongly simplify the sequence diversity and to significantly underweight the bacterial sequence number to obtain a clearer picture of the sequence diversity. We, therefore, extracted PDF sequences from completely sequenced genomes or from genomes for which sequencing was almost complete. A total of 262 sequences recapitulating the sequence diversity were finally aligned (Supplementary information). The four main PDF Types, including 3 subtypes in Type 1 (A/B/C), are clustered and colored in Fig. 1. As previously shown, in this updated phylogenetic tree, both *Vp*16T and *Vp*16C PDFs clustered in subtype 1B, as do the majority of bacterial PDFs. Type 1B PDFs are considered both plastid and bacterial PDFs, as this branch also includes all plant, algal and diatom plastid PDFs. Noticeably, with the increasing number of complete sequences now available, rather than the fragmented information from short reads of early metagenomics work, our early classification of marine viral PDF sequences tentatively assigned as Type 1^15^ had to be reconsidered and clustered into a new type - Type 4 - in the updated version of the phylogenetic tree (Fig. 1). Type 4 also includes various sequences from several apicomplexan eukaryotic parasites, such as *Toxoplasma gondii*, that were previously difficult to cluster. Finally, Type 3 is now enriched with a large variety of PDFs originating from all types of unicellular organisms, including prokaryotes and eukaryotes. As such, Type 3 is the most diverse in terms of organism diversity. This is unlike Type 2 PDFs, which are almost exclusively found in Gram-positive bacteria. Interestingly, Type 1A PDFs, previously believed to be only in eukaryotes, were also found in different bacteria.

### Phage *Vp*16 PDF is the smallest active PDF

**Figure 2:**
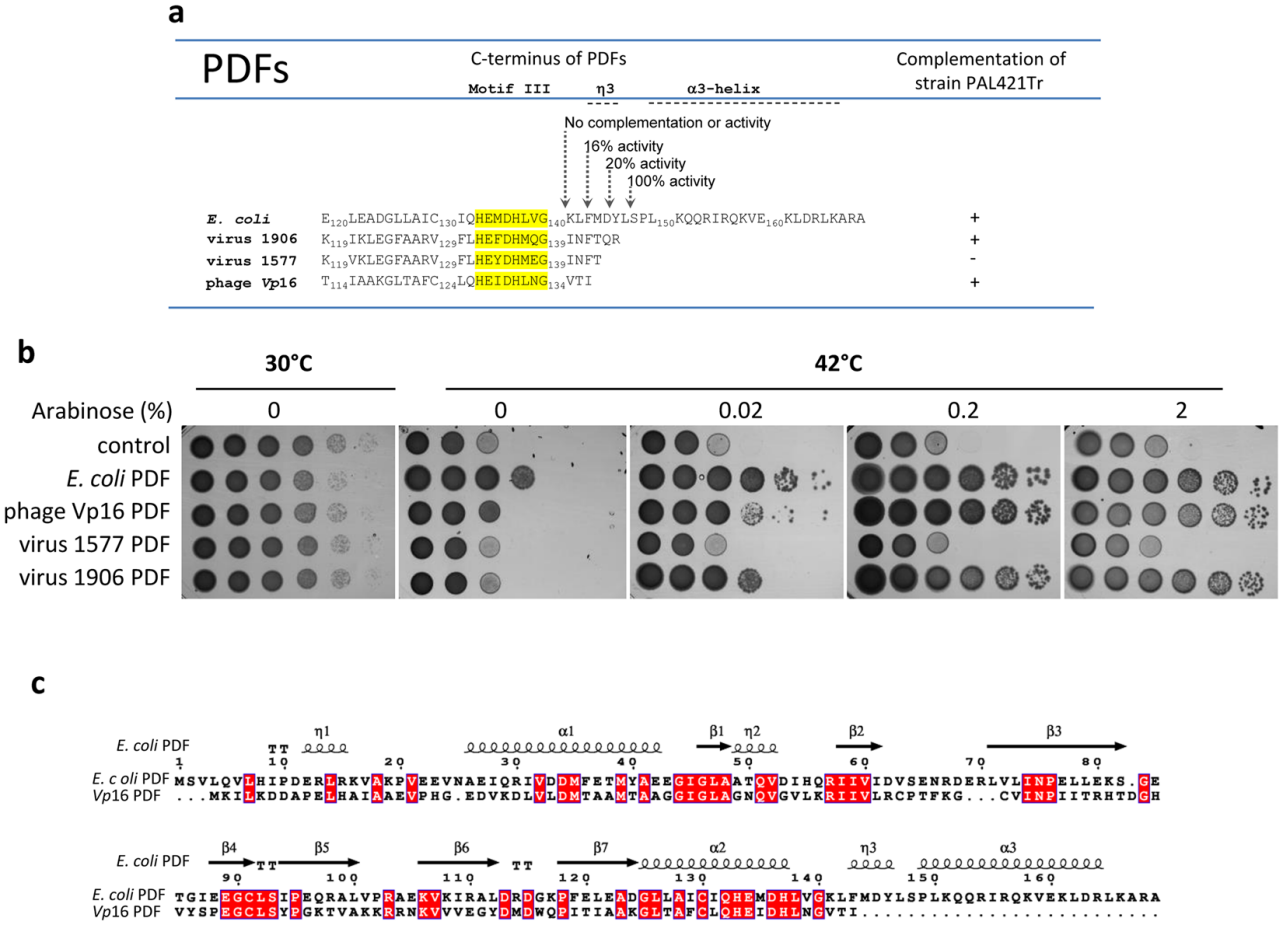
Complementation of strain PAL421Tr by PDFs with various length of their C-domain. pBAD plasmids encoding different PDFs (*E*. *coli*, *V*. *parahaemolyticus phage* and marine viral PDFs named: 1906 and 1577) were used to transform strain PAL421Tr at 30 °C. Strains were serially diluted and spotted in parallel at 42 °C on LB Petri dishes containing PDF expression inducer arabinose. Control corresponds to the empty cloning vector pBAD. (**a**) List of all used constructs, highlighting the corresponding C-terminus and results of the complementation. Various variants of *E*. *coli* PDF lacking the C-terminal region were previously constructed and activity tested *in vitro* and *in vivo* ^12^. (**b**) Image of the Petri dishes incubated at 30 °C or 42 °C at different concentration of arabinose. (**c**) sequence alignment between *E*. *coli* and *Vp*16 PDFs, realized with ESPript (http://endscript.ibcp.fr)^35^. Identical residues are shown on red boxes and secondary structures (α and 3_10_-helices, β-strands and turns) of *E*. *coli* PDF are indicated on top.

Although an increasing number of viral PDF sequences are regularly being identified, no data are available on the capacity of the corresponding proteins to function as PDF *in vivo*. Most viral PDFs, including marine viral PDFs and *Vp*16 PDF proteins, show high conservation in the three motifs that build the catalytic site; however, most contain shorter C-terminal extensions compared to the different active PDFs^15^. Because this C-terminal extension was shown to be important for PDF-ribosome binding and required for the *in vivo* deformylase activity of *E*. *coli* PDF (Fig. 2a and refs 11 and 12), we investigated the *in vivo* deformylase activity of several C-terminally truncated viral PDFs using the *def*-conditional strain PAL421Tr-pMAKdef^18^. Based on their relative distribution in the phylogenetic tree, we assessed (i) the representative members of marine viral PDFs – namely, those referred to as 1906, 1577 and 2750 in ref. 15, all three Type 4 PDFs, and (ii) the representative of the two almost identical *Vp*16 PDF proteins, which, unlike marine viral PDFs, clustered in Type 1B. The four sequences featured short C-domains of significantly reduced size (Fig. 2a). All viral PDF genes were cloned into the pBAD plasmid^19^, which features a tightly tunable arabinose-inducible promoter. We observed that both the *Vp*16 phage PDF and the marine viral 1906 PDF were active *in vivo*, as these enzymes allowed PAL421Tr growth under non-permissive conditions at the lowest arabinose concentrations (Fig. 2b). In contrast, no complementation was observed with marine viral 1577 and 2750 PDFs (Figs 2b and S1). Detailed analysis of the C-domains of these proteins (Fig. 2a,c) showed that the C-domain of the 1906 PDF is the longest compared to other viral PDFs, embracing the short η3 helix shown to be indispensable to fulfill deformylase activity of *E*. *coli* PDF (Fig. 2a and refs 11 and 12). Accordingly, a deletion in the 1906 PDF of the two last C-terminal residues located within the η3 helix, (1906QR), 1577 and 2750, did not lead to complementation (Figs 2a,b and S1). Instead, *Vp*16 PDF complemented PAL421Tr strain growth at non-permissive temperatures, although it did not contain residues expected to contribute to the crucial η3 helix of the C-domain (Fig. 2a). With only 137 residues *vs* an average of 165–175 residues for the prokaryotic PDFs, *Vp*16 PDF is thus unique among other PDFs, as it is the shortest PDF completely devoid of the C-domain (Fig. 2c) while exhibiting deformylase activity *in vivo*.

### Characterization of *Vp*16 PDF, a protein with high catalytic efficiency *in vitro* and with substrate specificity comparable to other PDFs

Enzymatic properties of purified *Vp*16 PDF were further determined *in vitro* with a formylated tripeptide (Fo-Met-Ala-Ser), which is used as a reference to compare various PDFs^20, 21^. *Vp*16 PDF showed a catalytic efficiency and *K*_m_ values for Fo-Met-Ala-Ser similar to that of other active PDFs (Table 1). However, it could not be excluded that *Vp*16 PDF has a particular substrate specificity to promote deformylation of either the proteins encoded by the phage genome *Vp*16T or to disfavor deformylation of some proteins of the host bacterium. Of note, the *in vitro* characterization of the deformylase activity of the PDF from cyanophage *Synechococcus* S-SSM7 using various formylated tetrapeptide derivatives of phage protein sequences has suggested a slight difference in the enzyme substrate specificity relative to the host enzyme^16^. To determine whether *Vp*16 PDF could specifically deformylate proteins encoded by the phage genome, we tested several formylated tripeptides derived from the N-terminal sequences of proteins encoded by the phage. Based on sequencing data, the genome of the phage *Vp*16T contains 64 ORFs^17^. We chose to test peptides Fo-Met-Pro-Ala and Fo-Met-Ser-Asn, corresponding to putative capsid and helicase N-termini, respectively. In addition, we chose peptides Fo-Met-Lys-Leu, Fo-Met-Thr-Thr and Fo-Met-Ala-Lys, corresponding to N-terminal sequences that are represented up to 3 times in the phage genome. The catalytic efficiencies of *Vp*16PDF and *E*. *coli* PDF for these peptides were determined and compared to reference peptide Fo-Met-Ala-Ser, which was set at 100%. The catalytic efficiencies of *Vp*16 PDF for Fo-Met-Lys-Leu and Fo-Met-Thr-Thr peptides were similar, while those of the Fo-Met-Pro-Ala and Fo-Met-Ser-Asn peptides were decreased (Table S1). Nevertheless, although slight differences appeared from these results, they were kinetically insignificant, and the same trends were obtained with the *E*. *coli* PDF (Table S1).

**Table 1:** Enzymatic properties of purified *Vp16* PDF and comparison with other PDFs.

PDF enzyme	*k*_cat_ (s^−1^)^a^	*K*_m_ (mM)^a^	*k*_cat_/*K*_m_ (M^−1^.s^−1^)^a^
Ni-*E*. *coli* PDF1B^a^	34–144	0.2–3.8	15,355–86,000
Ni-*T*. *thermophilus* PDF1B^c^	27 ± 3	2.3 ± 0.5	11,739 ± 2,500
*Synechocystis* PCC PDF1B^d^	150–250	1–2	88–313
Ni-*A*. *thaliana* PDF1B^b^	75 ± 15	5.6 ± 1.9	13,300 ± 1,500
Ni-*Vp*16 PDF1B	5.3–27.4	1–2.9	2,286–9448
*Synechococcus* phage S-SSM7 PDF4^d^	433–800	0.3–1.3	449–2,204
Zn-*A*. *thaliana* PDF1A^b^	22 ± 2	0.3 ± 0.1	88,000 ± 150
Ni*-H*. *sapiens* PDFIA^e^	0.26 ± 0.04	3.6 ± 0.9	72 ± 7
Ni-*B*. *stearotermophilus* PDF2^c^	1007 ± 191	4.1 ± 1.2	245,000 ± 20,000
Ni-*S*. *agalactiae* PDF2^f^	50 ± 3	1.2 ± 0.8	41,993
Ni-*P*. *falciparum* PDF3^g^	ND	ND	13,700 ± 1,000
Zn-*T*. *brucei* PDF3^h^	ND	ND	8

^a^Kinetic constants were determined using the coupled assay, as indicated in Materials and Methods, using substrates Fo-Met-Ala-Ser, Fo-Met-Lys-Leu, Fo-Met-Pro-Ala, Fo-Met-Ser-Asn, Fo-Met-Thr-Thr, Fo-Met-Ala-Lys when tested *Vp* 16 PDF1B and Ni-*E*. *coli* PDF1B.

^b^Data for *E*. *coli* and *Arabidopsis thaliana* PDF1Bs were taken from ref. 26.

^c^Data for *Thermus thermophilus* PDF1B and *Bacillus stearothermophilus* PDF2 were taken from refs 20, 28 and 37.

^d^Data were taken from ref. 16, substrates used: Fo-Met-Thr-Ser-Ile, Fo-Met-Leu-Ile-Ser, Fo-Met-Thr-Thr-Ala, Fo-Met-Ala-Lys-Lys, Fo-Met-Ala-Arg-Ile, Fo-Met- Ser-Arg-Val.

^e^Data for *Homo sapiens* PDF1A were taken from ref. 38.

^f^Data for *Streptococcus agalactiae* PDF2 were taken from ref. 28.

^g^Data for *Plasmodium falciparum* PDF3 were taken from ref. 27, substrate used: Fo-Met-Leu-p-nitroanilide.

^h^For *Trypanosoma brucei* PDF3 Data were taken from ref. 39.

ND, not determined.

Ni- or Zn indicates Ni^2+^- or Zn^2+^-containing PDFs.

**Figure 3:**
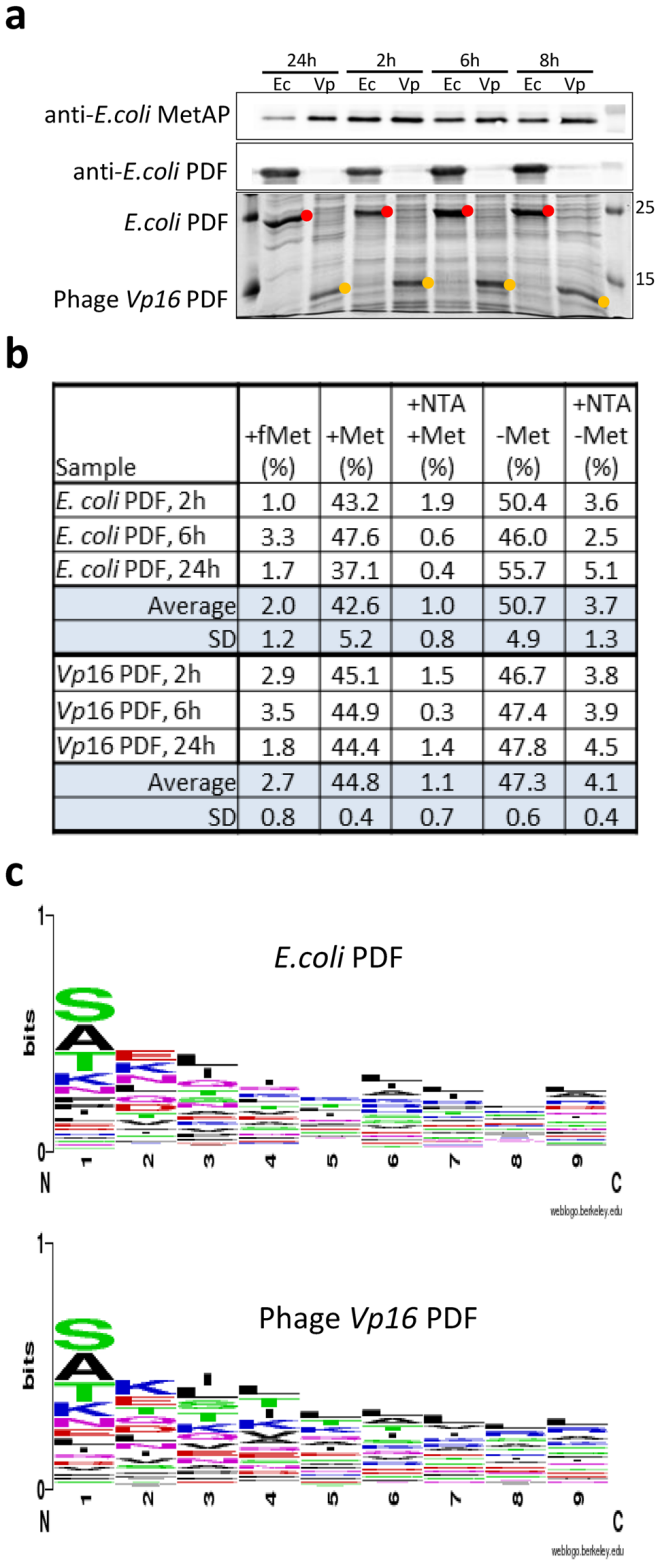
Large scale N-terminomics characterization for *in vivo* PDF substrate specificity determination. (**a**) PAL421Tr strain transformed with pBAD plasmids encoding *E*. *coli* or *Vp*16 PDFs were grown in LB liquid medium in the presence of 2% arabinose. At different time course growth rate, cultures were tested for the expression of *E*. *coli* MetAP and PDF (upper panels showing proteins detected by western blot with appropriate antibodies) or *Vp*16 PDF (lower panel showing culture lysates resolved on 12% SDS-PAGE stained with Comassie brilliant blue). Expressed *Vp*16 PDF protein is marked with yellow dots, expressed *E*. *coli* PDF is marked with red dots. The full-lenght blots and gel are presented in Supplementary Figure 3. (**b**) Samples from A were subjected to SILProNAQ methodology^1^ and distribution of the characterized N-terminal modifications was analyzed. (**c**) Protein N-terminus logo obtained for experimentally characterized N-termini from (**a**).

Next, a large scale N-termini proteomics characterization from culture cells expressing only *E*. *coli* or phage *Vp*16 PDF (Fig. 3a) revealed a similar distribution of the modifications affecting the N-termini along the growth profile (Fig. 3b). No difference was observed when the sequences of the identified N-termini undergoing deformylation were compared (Fig. 3b,c), further indicating that the two PDFs share an identical cleavage profile *in vivo*, *i*.*e*., show little preference, if any, for amino acids located beyond the amino-terminal formyl-methionine residue, as already described for *E*. *coli* PDF^1, 21, 22^.

### The crystal structure of *Vp*16 PDF reveals a classical PDF fold, in addition to a number of peculiarities

To gain insight into the properties of these novel viral PDFs, we determined the X-ray crystal structure of *Vp*16 PDF. The crystal structure of the protein could be solved by molecular replacement using the structure of *Pseudomonas aeruginosa* PDF without its C-terminal extremity as a search model. Two crystal forms of *Vp*16 PDF grown under different crystallization conditions were used. Both diffracted at a high resolution (1.7 to 1.5 Å) in space group *P3*_2_21 with nearly identical cell parameters and two molecules per asymmetric unit (Table S2). Superimposition of all chains showed very similar structures (r.m.s.d. of all of Cα < 0.5 Å). *Vp*16 PDF adopted an overall fold that is consistent with the Type 1B PDF family, and a search for structural homologs by DALI^23^ identified two Type 1B PDFs, namely, *Vibrio cholerae* (PDB code 3FWX, unpublished structure) and *E*. *coli* PDFs (PDB code 2AI8^24^), as the closest structural neighbors (sequence identity = 27 and 28%, r.m.s.d. = 1.05 Å and 1.08 Å, respectively). *Vp*16 PDF was found in a super-closed conformation, as evidenced by the delta aperture angle measured between Cys85, His131 and Ile41 (δ_ap_ = 41°–42°) (δ_ap_ is defined in ref. 25). Together, the data further validate the classification of *Vp*16 PDF as a Type 1B PDF (Fig. 1).

**Figure 4:**
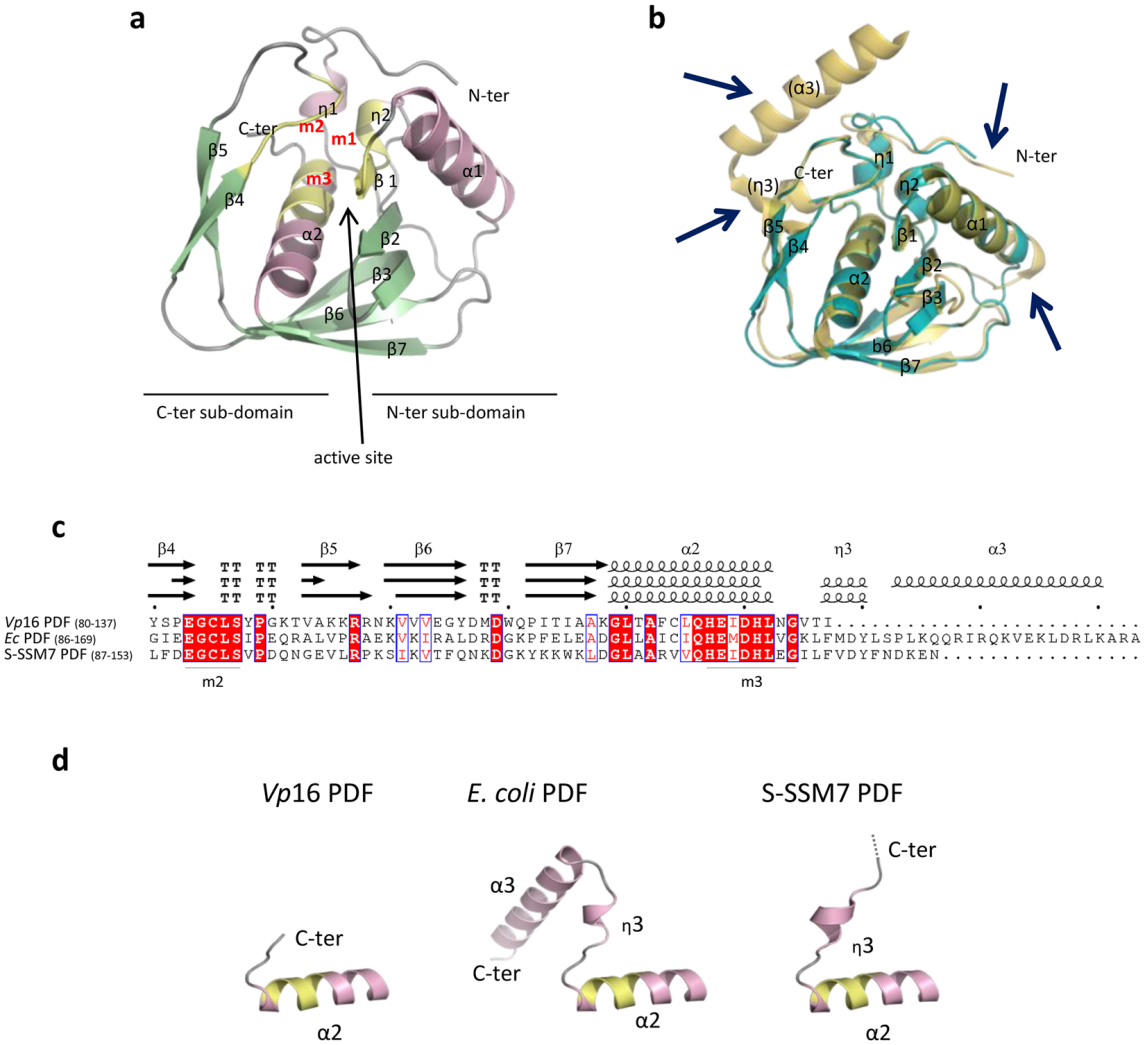
Crystal structure of *Vp*16 PDF. (**a**) *Vp*16 PDF ribbon diagram with α and 3_10_- helices in pink, β strands in green, showing the two sub-domains encircling the active site. The three consensus motifs (I, II and III) are highlighted in yellow. (**b**) Superimposition of *Vp*16 PDF with *E*. *coli* PDF structure. The main differences are indicated with arrows. (**c**) Comparison of C-terminal extremities of *Vp*16 PDF, *E*. *coli* PDF and S-SSM7 PDF was done by aligning sequences and secondary structures with ENDscript/ESPript tool (http://endscript.ibcp.fr)^35^. Identical residues are shown on red boxes and secondary structures (α and 3_10_-helices, β-strands and turns) are indicated on top. Motifs II and III (m2 and m3, respectively) are indicated. (**d**) Comparison of C-terminal folding. In contrast to *Vp*16 PDF, *E*. *coli* and S-SSM7 PDFs share the typical η3 3_10_-helix that follows conserved α2 helix. The flexible C-terminal extremity of S-SSM7 PDF is indicated with dotted lines. As in panel a, conserved motif III is highlighted in yellow.

The *Vp*16 PDF structure is composed of two α-helices (α1 and α2), two 3_10_-helices (η1 and η2) and seven β-strands (β1 to β7) organized into two sub-domains between which lies the active site (Fig. 4a). Although *Vp*16 PDF displays a classical PDF fold, several significant differences could be noted (Fig. 4b). First, *Vp*16 PDF displays a shorter N-terminus and an α1 helix reduced by one turn at its N-terminal extremity (Fig. 4b). The significance of the absence of one helical turn is currently unknown. Moreover, *Vp*16 PDF lacks the α3 helix usually found in Type 1B PDFs, as expected from sequence alignments (Fig. 4b,c). Unforeseen, *Vp*16 PDF is also devoid of the conserved η3 helix (Fig. 4c). Thus, *Vp*16 PDF lacks the two conserved structural elements of the C-domain. Interestingly, PDF from cyanophage S-SSM7, with a C-terminus slightly longer than that of *Vp*16 PDF (Fig. 4c), lacks the Type 1B typical α3 helix but displays the typical η3 helix, which is well-preserved in all PDFs determined thus far, including Type 1 and Type 2 (Fig. 4c).

**Figure 5:**
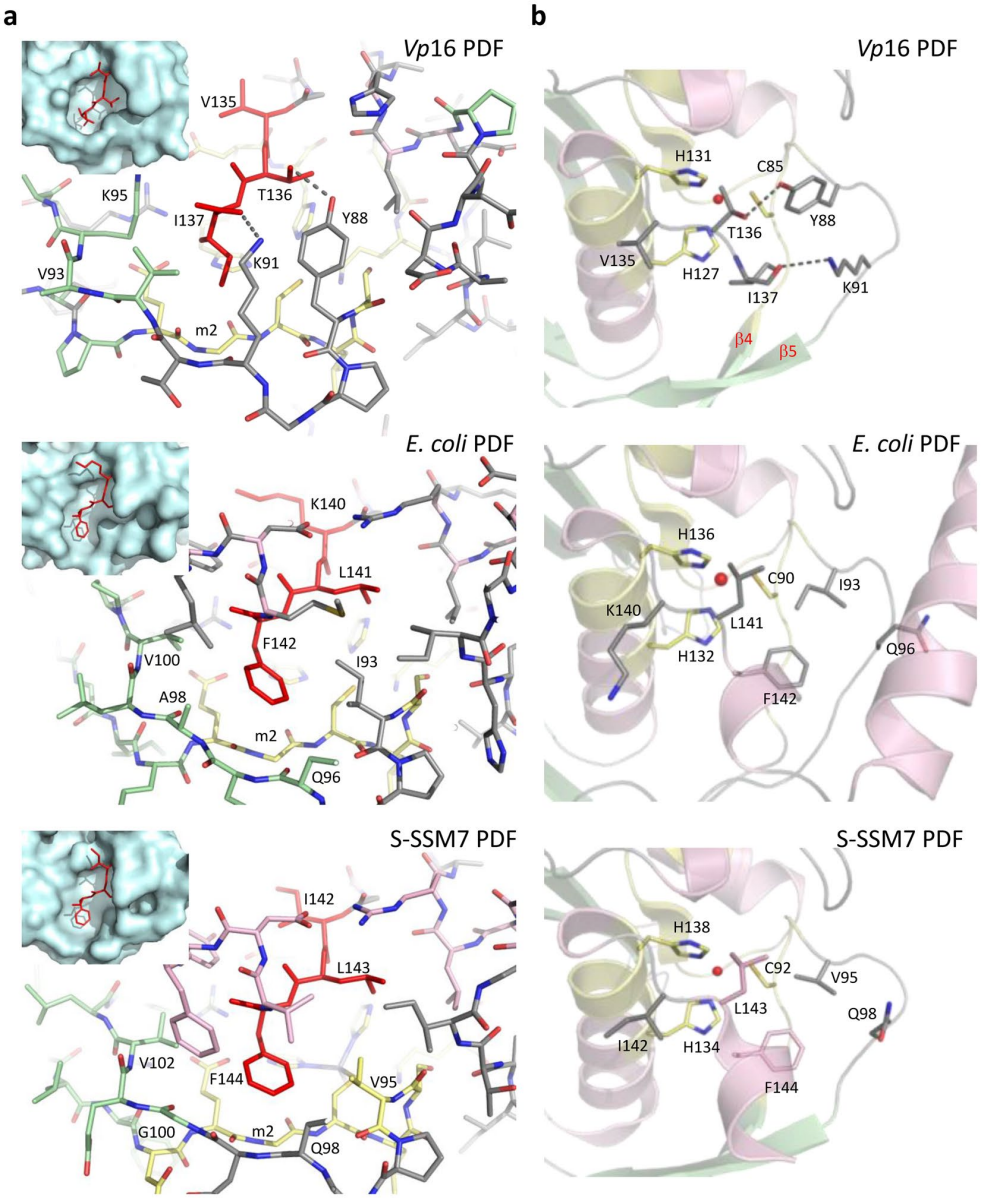
Comparison of interactions network at the C-terminal extremity of *Vp*16 PDF with the *E*. *coli* and S-SSM7 PDFs counterparts. The interactions network of *Vp*16 PDF C-terminal extremity was compared to that of *E*. *coli* and S-SSM7 PDFs. (**a**) The last three residues (V_135_T_136_I_137_) of *Vp*16 PDF are drawn in stick representation and colored in red. Other residues are shown in grey, with O and N atoms colored in red and blue, respectively. Hydrogen bond and salt bridge are represented by dotted lines. The same region is shown for *E*. *coli* and S-SSM7 PDFs. Inset: The hydrophobic pocket where *Vp*16 PDF Ile_137_ lies is highlighted through a surface representation of the protein in this region, in a slightly different orientation. The same region is also shown for *E*. *coli* and S-SSM7 PDFs. For more clarity, *E*. *coli* and S-SSM7 PDFs were truncated after counterpart residues of the last three residues of *Vp*16 PDF. (**b**) The proximity of *Vp*16 PDF C-terminal extremity with the active site (composed of residues His_127_, His_131_ and Cys_85_) is shown, and compared to the equivalent region in *E*. *coli* and S-SSM7 PDFs.

**Figure 6:**
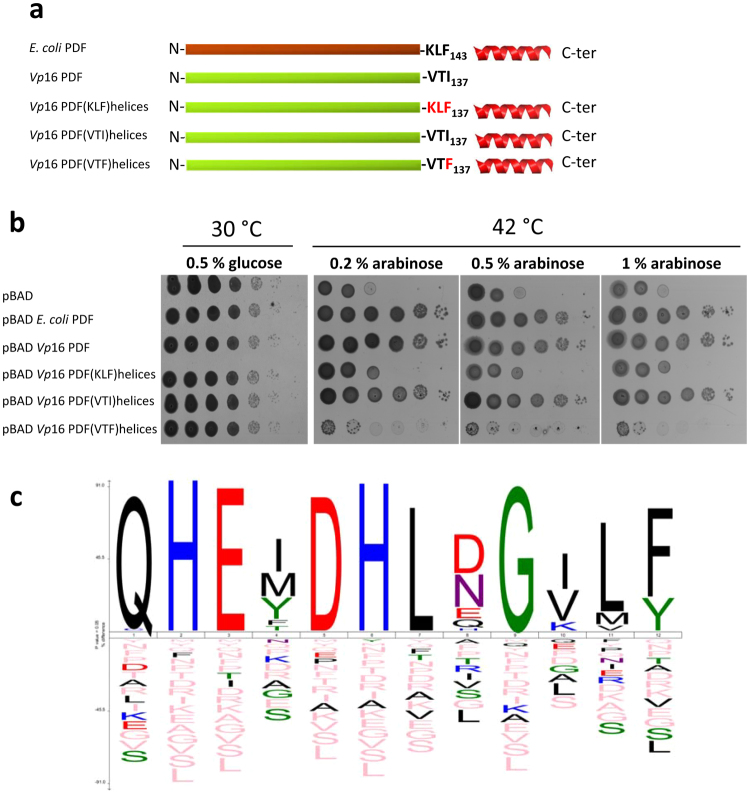
Complementation of strain PAL421Tr by different *Vp*16 PDF chimeras. pBAD plasmids encoding different PDF chimeras were used to transform strain PAL421Tr at 30 °C. Strains were serially diluted and spotted in parallel at 42 °C on LB Petri dishes containing PDF expression inducer arabinose. (**a**) Outline of used chimeras. (**b**) Image of the Petri dishes incubated at 30 °C and 42 °C at different concentration of arabinose. (**c**) The iceLogo 1.2 was used as the tool to display the data resulting from a collection of 237 distinct proteins^36^. Alignment starts with conserved motif III and the following 6 residues.

Further study of the C-terminal extremity of *Vp*16 PDF revealed that it is tightly bound to the globular core of the protein involving (i) a hydrogen bond made between the side chains of the penultimate residue, Thr_136_, and that of Tyr_88_ located immediately after motif II (m2, composed of residues 83–87) and (ii) a salt bridge between the C-terminal carboxyl group of the last residue, Ile_137_, and the side chain of Lys_91_, with both amino acids sitting in a structured loop between strands β4 and β5 (Fig. 5a). In addition, the side chain extremity of Lys_95_ is within hydrogen bonding distance of the carboxyl terminal of Ile_137_. The side chain of Ile_137_ is clamped by residues coming from β4 and β5 strands and their connecting loop, especially by Tyr_88_, Lys_91_, Val_93_ and Lys_95_ side chains (Fig. 5a and insert). These residues form a hydrophobic pocket where Ile_137_ is buried (Fig. 5a insert). Structural inspection of other Type 1B PDFs revealed that the overall network of interactions in the region of *Vp*16 PDF Ile_137_ does not occur in other PDFs (Fig. 5a). For instance, Thr_136_ and Ile_137_ of *Vp*16 PDF are usually replaced by two very apolar, bulkier residues in other PDFs regardless of the Type (see below and Fig. 6c), including in *E*. *coli* and S-SSM7 PDFs (Fig. 5a). More strikingly, the residues forming the Ile_137_ pocket of the *Vp*16 PDF are not conserved in *E*. *coli* and S-SSM7 PDFs (Fig. 5a), leading to two main differences in comparison to the *Vp*16 PDF. First, the hydrogen bond and the salt bridge observed in *Vp*16 PDF cannot occur in *E*. *coli* and S-SSM7 PDFs (Fig. 5a). Second, while the hydrophobic pocket around *Vp*16 PDF Ile_137_ appears rather rigid and constrained, the one found in *E*. *coli* and S-SSM7 PDFs is more open and agile. Of note, the special interaction network around the C-terminal extremity of *Vp*16 PDF is located in the vicinity of the three catalytic residues (Fig. 5b), evoking a possible link between the unusual extremity of *Vp*16 PDF and its biological function.

Several metal cations could be identified in the structure. Particularly, a zinc cation was identified within the active site, which was coordinated similarly to other PDF structures, *i*.*e*., by Cys of motif II, the two His of motif III and one water molecule.

### The crucial role of the last *Vp*16 PDF residue, Ile_137_, required for deformylase activity

The major sequence and structural differences between *Vp*16 PDF and *E*. *coli* PDF reside at the level of the C-terminal region, and we have shown that the η3 helix was necessary to preserve the complete deformylase activity of *E*. *coli* PDF^9^ but not *Vp*16 PDF (Fig. 2a). Therefore, to clearly identify the major determinant of the C-domain contributing to the full hydrolytic activity of *Vp*16 PDF, even without the η3 and α3 helices, we built a series of chimeras between the two proteins (Fig. 6a). All these chimeras were used to assess their deformylase activity both *in vivo* and *in vitro*. First, we generated a chimera made by *Vp*16 PDF to which we added the two C-terminal η3 and α3 helices of *E*. *coli* (*Vp*16 PDF(KLF)helices, Fig. 6a). As the last residue of *Vp*16 PDF (Ile_137_) corresponds to the first residue of the η3 helix of *E*. *coli* PDF (Fig. 2c), to ensure that this region would fold well in the form of a 3_10_-helix, we have substituted the last three residues of *Vp*16 PDF (Val_135_Thr_136_I_137_) with the *E*. *coli* counterpart motif Lys_135_Leu_136_Phe_137_. This chimera was unexpectedly found to be unable to complement the conditional PAL421Tr bacteria at non-permissive temperatures (Fig. 6b). In contrast, a chimera where the bacterial motif Lys_135_Leu_136_Phe_137_ was replaced with the phage residues Val_135_Thr_136_Ile_137_ (*Vp*16 PDF(VTI)helices, Fig. 6a) was able to fully complement the conditional PAL421Tr bacteria at non-permissive temperatures with an effectiveness comparable to *E*. *coli* PDF and *Vp*16 PDF (Fig. 6b). These data led us to a close inspection of the sequence surrounding the η3 helix of other known PDFs. The final *Vp*16 PDF dipeptides involving Thr_136_ and Ile_137_ are rather unusual and correspond generally to Leu or Met and Phe or Tyr residues, respectively, in other PDFs (Fig. 6c). We then constructed a new chimera in which we replaced the last Ile_137_ residue of *Vp*16 PDF with the equivalent Phe of *E*. *coli* (*Vp*16 PDF(VTF)helices, Fig. 6a). The only mutation in Ile_137_ of *Vp*16 PDF completely abolished the capacity of the protein to guarantee deformylase activity *in vivo* (Fig. 6b). This was in accordance with the enzymatic activity of the corresponding proteins measured *in vitro* (Table 2), highlighting the unexpected primary role of Ile_137_ in the deformylase activity of *Vp*16 PDF.

**Table 2:** Comparative enzymatic constants of *Vp*16 PDF and chimeras using Fo-Met-Ala-Ser as substrate.

PDF enzyme	*k*_cat_ (s^−1^)	*K*_*m*_ (mM)	*k*_cat_ /*K*_*m*_ (M^−1^.s^−1^)	Relative *k*_cat_/*K*_*m*_ (%)
*Vp*16 PDF1B	20 ± 1	2.3 ± 0.9	8,478 ± 2276	100
*Vp*16 PDF(KLF)helices	nm	nm	nm	nm
*Vp*16 PDF(VTI)helices	3.17	1	7,417 ± 6006	92
*Vp*16 PDF(VTF)helices	0.4	0.5	80	18
*E*. *coli* PDF1B	144	3.8	53,846 ± 21,667	100
*E*. *coli* PDF(KLF)Δhelices	77 ± 9.4	5.9 ± 2.1	13,125 ± 4,560	66
*E*. *coli* PDF(VTI)helices	26 ± 20	1.6 ± 1.3	19,316 ± 9,916	97
*E*. *coli* PDF(VTI)Δhelices	ND	ND	ND	ND
*E*. *coli* PDF(KLI)helices	41 ± 0.4	3.1 ± 0.11	13,189 ± 3,515	67

nm, not measurable.

ND, not determined.

To complete the above characterization, the constructs of mutant proteins of *E*. *coli* PDF were also carried out to mimic the structure of *Vp*16 PDF and to check the influence of the last three phage amino acids in the *E*. *coli* PDF enzyme in the presence and the absence of the η3 and α3 helices (Figure S2a). All these *E*. *coli* chimeras were able to complement strain PAL421Tr (Figure S2b). Nonetheless, the *E*. *coli* PDF enzyme lacking its η3 and α3 helices (PDF(KLF)Δhelices) was less efficient in complementing the PAL421Tr strain at low concentrations compared to all other constructs, including the corresponding proteins where Phe_143_ was substituted with the phage Ile_137_ (see PDF(VTI) helices, PDF(VTI)Δhelices and PDF(KLI) helices in Figure S2b). Taken together, all these data showed the crucial importance of Ile_137_ for efficient deformylase activity in the absence of the η3 and α3 helices.

## Discussion

The updated phylogenetic tree presented in this report, taking in account both the latest released PDF sequences, including those of viruses, and sequence diversity, allowed us to re-classify PDFs into four Types. In this new classification, phage PDFs clustered mainly into two Types: PDF1B and the new class, PDF4. Interestingly, PDF sequences retrieved in the GOS metagenomics database^15^, including phage S-SSM7^16^, clustered in the new class 4. Unlike previous studies, this phylogenetic tree shows that *Vp*16 PDF with other phage PDFs, clusters within the classical Type 1B PDF. Given this new classification, aside from the obvious but enthralling issue related to the purpose of why viruses express a PDF, the first query to solve was whether the respective encoded putative viral PDFs do display canonical deformylase activity. Indeed, despite strict conservation of the three motifs building the deformylase catalytic site, all identified viral PDF sequences, independent of the class to which they belong, show shorter C-termini compared to other PDFs^15^. Previous studies on *E*. *coli* PDF revealed that the C-domain can be trimmed, including the complete removal of α3 helix without affecting the activity of the enzyme *in vitro* or *in vivo* ^12^. However, too large of a deletion in the *E*. *coli* PDF C-terminus results in a decrease in activity until complete inactivation of the enzyme. Thus, a deletion after residues Leu_142_ or Met_144_ located just before or at the beginning of the η3 helix of *E*. *coli* PDF (Fig. 2a) causes a dramatic decrease in enzyme activity *in vitro*. A deletion beyond amino acid Gly_140_, in turn, contributes to a total loss of *E*. *coli* PDF activity^11, 12^. As the last amino acid of the only *in vitro* characterized viral PDF^16^, S-SSM7, corresponds to residue Gln_152_ in *E*. *coli* PDF (Fig. 4c), we could have assumed that despite its short size, S-SSM7 has a sufficient length to display full deformylase activity. However, the low uncommon enzymatic constant values obtained *in vitro* with both S-SSM7 and *Synechocystis* PCC PDFs (Table 2 in ref. 16), and particularly when compared to other known PDFs (Table 1), have left open the question regarding the deformylase activity of viral PDFs, independent of their affiliation. Of note, this uncertainty was much more marked for other shorter C-terminal viral PDFs and notably for *Vp*16 PDF, the last residue of which corresponds to residue Phe_143_ of *E*. *coli* PDF.

In this study, we show that several marine viral Type 4 PDFs and Type 1B *Vp*16 PDF do display deformylase activity *in vivo*. Moreover, *Vp*16 PDF displays *in vitro* deformylase activity comparable to other known active PDFs^26–30^. Clearly, with only 137 residues, *i*.*e*., 30 less than the average size of bacterial PDFs, *Vp*16 PDF is the smallest active PDF identified to date. This reduced size might reflect the need to take optimal advantage of the limited sequence information encoded in compact viral genomes, as already observed for other viral proteins (for review see ref. 31). On the other hand, it has been previously suggested that viral PDFs might exhibit specific distinct substrate specificity compared to bacterial PDFs^16^. Here, we provide evidence, using large-scale N-terminomics analysis, that *Vp*16 PDF has widespread substrate specificity similar to that of *E*. *coli* PDF.

What are the key elements allowing *Vp*16 PDF to ensure deformylase activity despite its extremely short C-terminus? Comparison of the structures of *Vp*16 and *E*. *coli* PDFs revealed that both PDFs display a classical PDF fold. However, the crystal structure of *Vp*16 PDF revealed, in addition, a number of differences. The most unexpected peculiarity is the absence of the η3 helix conserved in all characterized active PDFs, including PDF from cyanophage S-SSM7^16^. Interestingly, the two final residues of *Vp*16 PDF, Thr_136_ and Ile_137_, lying very close to the active site, are involved in a network of interactions unseen in any PDF thus far. Interestingly, Phe_142_ of *E*. *coli* PDF is highly conserved among the PDF family, due to the fact that a Phe is found in 76% of all analyzed sequences (it is replaced by a Tyr, which also contains an aromatic ring in less than 17% of the cases), and it is very unusual to find an Ile in this position (less than 2% of the cases). The Val/Ile/Lys-Leu-Phe/Tyr tripeptide appears to be by far the preferred consensus (Fig. 6c).

A close interdependency was observed between the dynamics of *Vp*16 PDF and its immediate environment (Fig. 5). We noticed indeed that the lateral chain of Ile_137_ of *Vp*16 PDF sits into a more tethered space than its counterpart residues in *E*. *coli* PDF (Phe_142_) and S-SSM7 PDF (Phe_144_). The large space around Phe_142_ in *E*. *coli* PDF and Phe_144_ in S-SSM7 PDF, compared to Ile_137_ from *Vp*16 PDF, confers a significant degree of freedom to the corresponding lateral chain, which might, in turn, contribute to unsettling the catalytic center and promote decreased activity. This mobility is most likely circumvented by the following C-domain residues building the η3 and α3 helices of the corresponding proteins. For instance, α3 helix in *E*. *coli* PDF is kept close to the rest of protein by a salt bridge between Asp_162_ and His_7_, which might restrain the environment around Phe_142_ and indirectly influence the optimal shape of the catalytic center. This might explain the low deformylase activity of the mutant *E*. *coli* PDF(KLF)Δhelices while pointing out the crucial role of Ile_137_ in *Vp*16 PDF. This was strengthened by a number of variants. The addition of the η3 and α3 helices of *E*. *coli* PDF at the C-terminus of *Vp*16 PDF, where the triplet Val_135_Thr_136_I_137_ was also substituted by the *E*. *coli* motif Lys_135_Leu_136_Phe_137_ or Val_135_Thr_136_Phe_137_, induces the complete loss of deformylase activity both *in vitro* and *in vivo*. In parallel, a simple substitution of the *E*. *coli* motif Lys_135_Leu_136_Phe_137_ with the *Vp*16 PDF triplet Val_135_Thr_136_I_137_ in the chimera *E*. *coli* PDF devoid of its C-terminal helices *E*. *coli* PDF(VTI)Δhelices improves the deformylase activity compared to the mutant *E*. *coli* PDF(KLF)Δhelices, particularly *in vivo*.

To conclude, characterization of the phage *Vp*16 PDF allows us to unexpectedly uncover the crucial role of the most unusual final amino acid in fully compensating for the 30 amino acid longer C-domain to ensure full deformylase activity.

## Materials and Methods

### PDFs cloning into the pBAD and pET16b vectors

Sequences of *Vibrio parahaemolyticus* phage PDF^17^ and representative members of marine viral PDFs: 1906, 1577, 2750^15^ were designed on the basis of data deposited in the metagenomics libraries. DNA sequences were synthesized and cloned into pBAD/*Myc*-HisA plasmid (Invitrogen) using BspHI and PstI cloning sites by GeneArt Company. Proteins expressed from final constructs resulted in amino acids sequences described in Table S3. Sequence of 1906 PDF was further modified by deletion of the last two amino acids Q144 and R145. *Vp*16 PDF was subcloned from pBAD/*Myc*-HisA plasmid into pET16b using NcoI and XhoI cloning site.

### *V*. *parahaemolyticus* phage PDF expression and purification

*Vp*16 PDF purification has been performed using previous protocols with several modifications (Supplementary information). Identity of purified *Vp*16 PDF protein was confirmed by MALDI-TOF analysis with 88% of sequence coverage.

### Complementation test

The *def*-conditional strain PAL421Tr-pMAKdef (*fms*_1, *galK*,*rpsL*, *recA*56, *srl*-300::Tn10), also known as PAL421Tr, with inactivated fms gene on the chromosome and the wild-type allele on the thermosensitive pMAK vector has been described in ref. 18. pBAD plasmid encoding PDF proteins were transformed into the PAL421Tr *E*. *coli* strain at 30 °C. Next day strains were streaked out in parallel at 42 °C on LB dishes containing different concentrations of the PDF inducer arabinose. Negative control corresponded to the empty cloning vector pBAD.

### N-terminal proteomic analysis

Proteomic analyses were performed essentially as previously described^1^. Further details are provided in the Supplementary information.

### Crystallization and structure determination

Crystallization conditions of apo *Vp*16 PDF were screened by a robot using commercial kits (JCSG+ suite and PEGs suite from QIAgen, and Precipitant Synergy screen from Jena Bioscience). Crystallization conditions were obtained and manual optimizations were then carried out at 19 °C with drops of 1:1 mixture of protein (5 mg/mL) and reservoir solution. Best crystals were obtained in 38% MPD, 100 mM CAPS pH 10.5 (form I) and 28% PEG-1,000, 100 mM sodium acetate pH 5.5 (form II). Prior to data collection, manually reproduced crystals were harvested, transferred to a cryoprotectant solution (mother liquor containing 20% of glycerol) and flash frozen in liquid nitrogen. Data collection and processing are described in the Supplementary information.

## Electronic supplementary material

Supplemental information
