# Supplementary material for: The C-terminal residue of phage Vp16 PDF, the smallest peptide deformylase, acts as an offset element locking the active conformation
Source: Sci Rep. 2017 Sep 8;7:11041. doi: 10.1038/s41598-017-11329-3 (PMC5591237; doi:10.1038/s41598-017-11329-3)
Supplement: Supplementary file 1 — Supplemental information [file 41598_2017_11329_MOESM1_ESM.pdf]

SUPPLEMENTAL INFORMATION FOR

The C-terminal residue of phage *Vp*16 PDF, the smallest peptide deformylase, acts as an offset element locking the active conformation

Renata Grzela, Julien Nusbaum, Sonia Fieulaine, Francesco Lavecchia, Willy V. Bienvenut, Cyril Dian, Thierry Meinnel[#] & Carmela Giglione[#]

[#]Corresponding authors E-mail carmela.giglione@i2bc.paris-saclay.fr or thierry.meinnel@i2bc.paris-saclay.fr

This PDF file includes:

Materials and Methods

Supplementary References

Supplementary Figure Legends

Supplemental Tables

Supplementary Figures

MATERIALS AND METHODS

Materials

All solvents and chemicals were purchased from SDS and Aldrich, respectively.

V. parahaemolyticus phage PDF expression and purification

Rosetta2(DE3)pLysS were transformed with pET16b plasmid containing *Vp*16 PDF gene. Protein expression was induced with 0.5 mM IPTG for 3h at 37°C. For the crystal structure determination *Vp*16 PDF was purified as previously described [1]. For the enzymatic characterization, the cells were resuspended in buffer A (50 mM MES-KOH pH 4 and 80 mM $NiCl_2$). The supernatant was loaded to SP-Sepharose column equilibrated with buffer A. Bound proteins were eluted with buffer B (50 mM MES-KOH pH 4 and 80 mM $NiCl_2$, 1M KCl). Because a high purity, no second step of purification was necessary and purified protein was directly dialyzed overnight at 4°C against buffer A containing 55% glycerol and stored at -20°C.

Cloning, expression and purification of *E. coli* PDF and chimeras

E. coli PDF and *E. coli* PDF(KLF)Δhelices were cloned in pBAD and pET-22b(+) as previously reported [2;3]. Site-directed mutagenesis to create the different chimeras was carried out in the wild type version of *E. coli* PDF or *Vp*16 PDF present in both pBAD and pET-22b(+) plasmids by using the QuickChange Site-Directed Mutagenesis Kit (Stratagene). The C-terminal deleted versions of *E. coli* PDF and corresponding mutants were generated from the corresponding full length versions in which a double stop was inserted after the codon corresponding to the 144th amino acid.

*Vp*16 PDF(KLF)helices DNA sequence was optimized, synthesized and cloned into pBAD/*Myc*-HisA plasmid (Invitrogen) using NcoI and XhoI cloning sites by GeneArt Company. The other *Vp*16 PDF chimeras were obtained by site-directed mutagenesis of *Vp*16 PDF(KLF)helices DNA sequence into pBADMyc-HisA plasmid. All corresponding *Vp*16 PDF chimeras were sub-cloned in pET-16b plasmid (Novagen) for protein purification. Primers used are described in **Table S3**. Proteins expressed from final constructs resulted in amino acids sequences described in **Table S4**. Purification of chimeras has been performed as the corresponding wild type protein.

Enzymology

PDF enzymatic activity was measured by a spectrophotometric assay. The formate released by the deformylation reaction is used by FDH to convert NAD in NADH, the production of which being measured over time at 340 nm and controlled temperature (37°C). The 200µL reaction mixture contained 50 mM HEPES-NaOH pH 7.5, 12 mM NAD^+, 4.5 U/mL FDH, 1 mM $NiCl_2$ and PDF enzyme (either 100 nM *Vp*16 PDF or 10 nM *E. coli* PDF) previously diluted in 50 mM MES-KOH pH 4.0, 80 mM $NiCl_2$ for *Vp*16 PDF or 50 mM HEPES-NaOH pH 7.5, 0.1 mg/mL BSA for *E. coli* PDF. The reaction was started by addition of 1-6 mM Fo-peptides as indicated in the legend of Figure. Kinetics parameters (k_{cat}, K_m) were derived from iterative non-linear least square fits using the Michaelis-Menten equation based on the experimental data (Sigma-Plot, Kinetics module).

*Vp*16 PDF inhibition was performed as previously described [4, 5].

Immunological Methods

Rabbit polyclonal antibodies raised against full length *E. coli* MetAP and *E. coli* PDF were used at 1:5000 dilution for Western blot analysis as previously described [6].

N-terminal proteomic analysis

The PAL421Tr was transformed at 30°C with pBAD plasmid expressing *Vp*16 PDF or *E. coli* PDF in the presence of ampicillin. Next day 10 mL cultures were inoculated with bacterial colony from the plate and grown for 24h in the presence of ampicillin and 0.2% arabinose at 37°C. After 24h, cultures were diluted to the OD600=0.05 and cultured at 30°C in the presence of 0.1% of arabinose for cells expressing *E. coli* PDF and 0.2% for *Vp*16 PDF to equalize the level of protein expression. Cells (4.6 x 10^{10}) were harvested and collected by centrifugation (4°C, 3300g, 30 min) at time points 2, 6, 8 and 24h. Cells were disrupted by grinding (MM300 grinder, Qiagen) in 50 mM Hepes-NaOH pH 8, 25 mM Ascorbic Acid, 25 mM L-Cystein and one tablet of antiprotease inhibitor cocktail (Roche). After centrifugation at 21,000 g for 30 min at 4°C, protein concentration in the supernatant was determined by Bradford protein assay.

For N-termini enrichment samples were prepared as previously described (Bienvenut et al., 2012). 1 mg of protein was denatured and reduced followed by cysteine alkylation with iodoacetamide. After cold acetone precipitation, proteins were resuspended in 50 mM NH_4HCO_3 and digested by 1/100 (w/w) of TPCK treated porcine trypsin (Sigma-Aldrich) for 1.5h at 37°C, twice. Peptides were desalted with Sep-Pak columns and the retained material was eluted with 80% acetonitrile (ACN), 0.1% TFA and then evaporated to dryness. The collected material was resuspended in Strong Cation eXchange (SCX) LC buffer (5mM KH_2PO_4, 30% ACN and 0.05% formic acid) and injected into Alliance HPLC system using a fluorimeter detector (Waters) equipped with polysulfoethyl A column (200 x 2.1 mm, 5 µm 200 Å; PolyLC, Colombia, MD). Peptides were eluted with a KCl gradient (SCX-LC buffer B: 350 mM KCl in SCX-LC buffer A; 0-5 min, 0% B; 15-40 min, 5-26% B; 40-45 min, 26-35% B). Fractions were collected every 2 min for 40 min and the solvent was evaporated to

dryness before storage at -20°C. Fractions eluted from SCX columns with retention times of 3 to 22 min were analyzed as previously described [7] with an Easy Nano-LC II (Thermo Scientific) coupled to a LTQ-Orbitrap™ Velos (Thermo Scientific).

Crystallography: data collection and processing

Diffraction data were collected on single crystals at 100 K on FIP-BM30A and PROXIMA1 beamlines at ESRF (Grenoble, France) and SOLEIL (Gif-sur-Yvette, France), respectively. Data were processed and scaled with XDS package [8]. Statistics are summarized in **Table S2**. The crystal structure of *Vp*16 PDF crystallized in form I was solved by molecular replacement with PHASER [9] using a C-terminally truncated version of the *Pseudomonas aeruginosa* PDF (PDB code 1LRY [10]) as the starting model. Structures of protein crystallized in form II was solved by rigid-body refinement in REFMAC [11]. Manual model building and refinement were done with TURBO-FRODO [12] and REFMAC, [11] respectively, yielded the final models that were validated by PROCHECK [13]. Refinement statistics are detailed in **Table S2**. Figures were generated using PyMOL (http://www.pymol.org).

Inspection of the density maps revealed the presence of several high electron density spots in both models, which could reasonably be interpreted as metal ions. In order to identify the nature of these ions, we performed X-ray fluorescence experiments on the crystal form II, which revealed the presence of two different metal ions, zinc and nickel. Anomalous difference maps calculated for each metal allowed us to locate zinc ions within the active site and nickel ions at the surface of the protein.

List of the 262 sequences selected to represent PDF sequence diversity and to construct the new phylogenetic tree

Type 1B

Halococcus-hamelinensis (archaea); Roseobacter-sp-MED193; Rhodobacterales-bacterium-HTCC2083; Roseobacter-denitrificansRoseovarius-217; Magnetospirillum-magneticum-AMB1; Magnetospirillum-magnetotacticum-MS1; Phaeobacter-gallaeciensis-BS107; Sinorhizobium-meliloti1/2 ;Agrobacterium-tumefaciens1/2; Caulobacter-phageCcr; Colossus Sinorhizobium-meliloti2/2; Agrobacterium-tumefaciens2/2; Vibrio-cholerae1/2; Vibrio-parahaemolyticus1/2; Enterobacter-aerogenes; Escherichia-coli; Haemophilus-influenzae; Neisseria-gonorrhoeae; Chromobacterium-violaceum; Physcomitrella-patens1/3; Pseudomonas-aeruginosa1/2; Methylococcus-capsulatus2/2; Xanthomonas-oryzae2/2; Vibrio-cholerae2/2; Vibrio-parahaemolyticus2/2; Rickettisae-prowazekii; Rickettsia-conorii2/3; Ehrlichia-canis1/1; Rickettsia-conorii3/3; Clostridium-sordellii1/2; Peptoclostridium-difficile2/2; Clostridium-tetani; Clostridium-acetobutylicum1/2; Clostridium-botulinum2/3; Clostridium-clostridioforme2/2; Clostridium-perfringens2/2; Candidatus-arthromitus; Clostridium-botulinum1/3; Clostridium-beijerinckii1/2; Clostridium-acetobutylicum2/2; Peptoclostridium-difficile1/2; Bacillus-oceanisediminis1/3; Geobacillus-stearothermophilus1/2; Bacillus-cereus1/2; Bacillus-subtilis2/2; Enterococcus-faecalis1/2; Listeria-monocytogenes1/2; Helicobacter-pylori; Myxococcus-xanthus3/3; Mycobacterium-tuberculosis; Mycobacterium-leprae; Mycobacterium-smegmatis; Nocardia-vulneris3/4; Rhodococcus-wratislaviensis1/2; Corynebacterium-diphtheriae1/2; Corynebacterium-doosanense2/2; Corynebacterium-amycolatum1/2; Nocardia-vulneris1/4; Rhodococcus-wratislaviensis2/2; Streptomyces-avermitilis2/3; Streptomyces-avermitilis1/3; Gardnerella-vaginalis1/2; Bifidobacterium-longum1/3; Thermobifida-fusca; Nocardia-vulneris4/4; Corynebacterium-amycolatum2/2; Corynebacterium-diphtheriae2/2; Corynebacterium-doosanense1/2; Capronia-semi-immersa Cladophialophora-carrionii ; Cladophialophora-psammophila; Propionibacterium-humerusii; Propionibacterium-acnes; Tropheryma-whipplei-strTwist; Aquifex-aeolicus; Hydrogenobacter-thermophilus-TK6; Ricinus-

communis1B; Populus-trichocarpa1B; Vitis-vinifera1B; Glycine-max1B; Medicago-truncatula1B; Lycopersicon-esculentum1B; Arabidopsis-thaliana1B; Hordeum-vulgare1B; Oryza-sativa1B; Micromonas-pusilla; Micromonas-sp.RCC299-1B; Ostreococcus-lucimarinus1B; Chlamydomonas-reinhardtii1B; Galdieria-sulphuraria; Thalassioria-pseudonana2/2-diatom; Cyanidioschyzon-merolae-rhodophyte; Thalassiosira-pseudonana1/2-diatom; Thermotoga-spRQ2; Thermotoga-naphthophila; Thermotoga-maritima-MSB8; Thermotoga-petrophila-RKU1; Thermotoga-neapolitana-DSM4359; Petrotoga-mobilis; Bigelowiella-natans1B; Plasmodium-vivax ;Plasmodium-knowlesi; Plasmodium-falciparum; Plasmodium-yoelii; Acanthamoeba-castellanii1/2 amoebozoa; Psychroflexus-torquis-ATCC700755; unidentified-eubacterium-SCB49; Polaribacter-irgensii-23P; Flavobacterium-seoulense; Sphingobacterium-spiritivorum-ATCC33861; Algoriphagus-sp-PR1; Microscilla-marinaBacteroides-sp.D1; Bacteroides-ovatusATCC8483; Bacteroides-thetaiotaomicron-VPI-5482; Pseudomonas-gingivalis1/2; Chlorobium-tepidum ;Cyanothece-sp.PCC7424; Crocosphaera-watsoniiWH8501; Synechocystis-sp-PCCCalothrix-sp.; Acaryochloris-marina-MBIC11017; Gloeobacter-violaceus; Prochlorococcus-marinus-strMIT9215; Prochorococcus-marinus; Synechococcus-spRS9917; Borrelia-burgdorferi ; Treponema-pallidum; Myxococcus-xanthus2/3.

Type 1C

Streptobacillus-moniliformis-DSM1211; Leptotrichia-buccalis; Sebaldella-termitidis-ATCC33386; Methanocorpusculum-bavaricum1/2; Methanocorpusculum-labreanum-Z2/2; Methanocorpusculum-bavaricum2/2; Methanocorpusculum-labreanum-Z1/2; Methanomicrobium-mobile; Deinococcus-radiodurans.

Type 1A

Methylococcus-capsulatus1/2; Myxococcus-xanthus1/3; Bos-taurus; Muntiacus-muntjak-vaginalis-deerCanis-familiaris; Homo-sapiens; Pan-troglodytes-chimpanzee; Mus-

musculusRattus-norvegicus; Gallus-gallus; Meleagris-gallopavo turkey; Tetraodon-nigroviridis; Fugu-rubripes; Gasterosteus-aculeatus; Oryzias-latipes-Japanese-medaka; Danio-rerio; Oncorhynchus-mykiss; Xenopus-tropicalis; Ixodes-scapularis-tick-arachnide; Boophilus-microplus-tick-arachnide; Strongylocentrotus-purpuratus-echynoderm; Drosophila-melanogaster2/2; Drosophila-melanogaster1/2; Anopheles-gambiae; Aedes-aegypti yellow; Oncometopia-nigricans; Apis-melliferabee; Hydra-magnipapillata2/2; Hydra-magnipapillata1/2; Batrachochytrium-dendrobatidis; Spizellomyces-punctatus; Physcomitrella-patens2/3; Physcomitrella-patens3/3; Lycopersicon-esculentum1A; Arabidopsis-thaliana1A; Oryza-sativa1A; Triticum-aestivum1A; Chlamydomonas-reinhardtii1A; Ostreococcus-lucimarinus1A; Streptomyces-coelicolor1; Streptomyces-avermitilis3/3; Nocardia-vulneris2/4; Gardnerella-vaginalis2/2 ; Bifidobacterium-longum2/3; Xanthomonas-oryzae1/2; Pseudomonas-aeruginosa2/2.

Type 4

Cyano-phage-GOS-JCVI-1502.1906; Cyano-phageGOS-JCVI-1149-1577; Cyano-phageP-RSM6; Cyano-phageGOS-JCVI-1906; Stenotrophomonas-phage-vB; SmaS-DLP 6; Caulobacter-phageCr30; Synechococcus-phageACG-2014f; Synechococcus-phage S-SSM7 ; Ehrlichia-canis2/2; Hammondia-hammondi; Toxoplasma-gondii; Neospora-caninum.

Type 3

Vibrio-phage-henriette-12B8; Methanoculleus-marisnigri; Candidatus-Methanoperedens-nitroreducens; Methanosarcina-soligelidi; Methanobacterium-formicicum; Methanobacterium-thermoautotrophicum; Methanobrevibacter-filiformis; Trypanosoma-cruzi2/2; Trypanosoma-brucei2/2; Leishmania-major2/2; Trypanosoma-cruzi1/2; Trypanosoma-brucei1/2; Leishmania-major1/2; Thermoplasmatales-archaeon; Streptococcus-dysgalactiae2/2; Streptococcus-pyogenes2/2; Streptococcus-uberis2/2; Streptococcus-thermophilus2/2; Streptococcus-agalactiae2/2; Streptococcus-suis2/2; Streptococcus-

pneumoniae2/2; Clostridium-botulinum3/3; Clostridium-beijerinckii2/2; Clostridium-sordellii2/2; Erysipelotrichaceae-bacterium2/2; Bifidobacterium-longum3/3; Lactobacillus-helveticus2/2; Rickettsia-conorii1/3; Staphylococcus-simiae2/2; Staphycoccus-aureus2/2; Clostridium-perfringens1/2; Paramecium-tetraurelia2/2; Nostoc-punctiforme; Malawimonas-jakobiformis-mitochondrial-cristae; Blastocystis-homini-diatom; Clostridium-clostridioforme1/2 ; Candidatus-thorarchaeota archaea; Lokiarchaeum-sp archaea; Sawyeria-marylandensis; Polysphondylium-pallidum; Acytostelium-subglobosum; Dictyostelium-discoideum; Acanthamoeba-castellanii2/2; Paramecium-tetraurelia1/2.

Type 2

Streptococcus-uberis1/2; Streptococcus-thermophilus1/2; Streptococcus-agalactiae1/2; treptococcus-pneumoniae1/2; Streptococcus-dysgalactiae1/2; Streptococcus-suis1/2; Enterococcus-faecalis2/2; Listeria-monocytogenes2/2; Bacillus-subtilis1/2; Geobacillus-stearothermophilus2/2; Bacillus-oceanisediminis3/3; Bacillus-cereus2/2; Staphylococcus-simiae1/2; Staphycoccus-aureus1/2; Bacillus-oceanisediminis2/3; Lactobacillus-helveticus1/2; Erysipelotrichaceae-bacterium1/2; Mycoplasma-pneumoniae; Phialophora-attae

SUPPLEMENTARY REFERENCES

1. Ragusa S, Mouchet P, Lazennec C, Dive V, Meinnel T. Substrate recognition and selectivity of peptide deformylase. Similarities and differences with metzincins and thermolysin. *J Mol Biol* **289**, 1445-1457 (1999).

2. Fieulaine S, Desmadril M, Meinnel T, Giglione C. Understanding the highly efficient catalysis of prokaryotic peptide deformylases by shedding light on the determinants specifying the low activity of the human counterpart. *Acta crystallographica Section D, Biological crystallography* **70**, 242-252 (2014).

3. Serero A, Giglione C, Meinnel T. Distinctive features of the two classes of eukaryotic peptide deformylases. *J Mol Biol* **314**, 695-708 (2001).

4. Fieulaine S, *et al.* Trapping conformational states along ligand-binding dynamics of peptide deformylase: the impact of induced fit on enzyme catalysis. *PLoS Biol* **9**, e1001066 (2011).

5. Boularot A, *et al.* Discovery and refinement of a new structural class of potent peptide deformylase inhibitors. *J Med Chem* **50**, 10-20 (2007).

6. Adam Z, Frottin F, Espagne C, Meinnel T, Giglione C. Interplay between N-terminal methionine excision and FtsH protease is essential for normal chloroplast development and function in Arabidopsis. *Plant Cell* **23**, 3745-3760 (2011).

7. Bienvenut WV, Giglione C, Meinnel T. Proteome-wide analysis of the amino terminal status of Escherichia coli proteins at the steady-state and upon deformylation inhibition. *Proteomics* **15**, 2503-2518 (2015).

8. Kabsch W. Integration, scaling, space-group assignment and post-refinement. *Acta crystallographica Section D, Biological crystallography* **66**, 133-144 (2010).

9. McCoy AJ, Grosse-Kunstleve RW, Storoni LC, Read RJ. Likelihood-enhanced fast translation functions. *Acta Crystallogr D Biol Crystallogr* **61**, 458-464 (2005).

10. Guilloteau JP, *et al.* The crystal structures of four peptide deformylases bound to the antibiotic actinonin reveal two distinct types: a platform for the structure-based design of antibacterial agents. *J Mol Biol* **320**, 951-962 (2002).

11. Murshudov GN, *et al.* REFMAC5 for the refinement of macromolecular crystal structures. *Acta crystallographica Section D, Biological crystallography* **67**, 355-367 (2011).

12. Roussel A, Cambillau C. TURBO-FRODO. In: *Silicon Graphics geometry partners directory* (ed^(eds Graphics S) (1989).

13. Laskowski RA, MacArthur MW, Moss DS, Thornton JM. PROCHECK-a program to check the stereochemical quality of protein structures. *J Appl Cryst* **26**, 283-291 (1993).

SUPPLEMENTARY FIGURE LEGENDS

Figure S1 Complementation of strain PAL421Tr by different PDFs. pBAD plasmids encoding different PDFs (*E. coli*, *Vibrio parahaemolyticus* phage and marine viral PDFs named: 1906, 1906QR deleted version, the last two amino acids, of 1906, and 2750) were used to transform strain PAL421Tr at 30°C. Strains were streaked out in parallel at 42°C on

LB Petri dishes containing PDF expression inducer arabinose (0.1%). pBAD corresponds to the empty cloning vector used as control.

Figure S2 Complementation of strain PAL421Tr by different *E. coli* PDF chimeras. pBAD plasmids encoding different PDF chimeras were used to transform strain PAL421Tr at 30°C. Strains were serially diluted and spotted in parallel at 42°C on LB Petri dishes containing PDF expression inducer arabinose. **a**) Outline of used chimeras. **b**) image of the Petri dishes incubated at 30°C and 42°C at different concentration of arabinose.

Figure S3 Full-lenght gel and blots of Figure 3a are presented.

SUPPLEMENTAL TABLES

Table S1. Comparative enzymatic constants of *Vp*16 PDF and *E. coli* PDF for different formylated tripeptides.

Peptide	*Vp*16 PDF				*E. coli* PDF			
	K_m (mM)	k_{cat} (sec^{-1})	k_{cat} / K_m (M^{-1} s^{-1})	k_{cat} / K_m (%)	K_m (mM)	k_{cat} (s^{-1})	k_{cat} / K_m (M^{-1} s^{-1})	k_{cat} / K_m (%)
**Fo-Met-Ala-Ser**	2.3	20	8,478	100	2.59	57	22,800	100
**Fo-Met-Lys-Leu**	2.9	27.4	9,448	111	0.29	344	86,000	377
**Fo-Met-Pro-Ala**	1.4	5.3	3,711	44	5.1	82	16,080	70
**Fo-Met-Ser-Asn**	12	27.4	2,286	27	5.8	89	15,355	67
**Fo-Met-Thr-Thr**	1.0	9.0	8,950	105	1.4	70	49,366	216

The catalytic efficiency for the Fo-Met-Ala-Ser was taken as 100% activity.

Table S2. Crystallographic data and refinement statistics

	Form I	Form II
Ligand	---	PEG-1,000
Data collection		
Wavelength (Å)	0.97978	0.98011
Resolution range (Å)	50.0-1.7 (1.80-1.70)	50.0-1.5 (1.54-1.50)
Space group	P 3_2 2 1	P 3_2 2 1
Unit cell		
a, b, c (Å)	64.73, 64.73, 128.53	64.26, 64.26, 125.69
α, β, γ (°)	90, 90, 120	90, 90, 120
Unique reflections	35064	48793
Redundancy	9.0 (8.9)	4.3 (4.4)
Completeness (%)	99.8 (99.8)	99.7 (99.5)
I/σ	20.33 (3.28)	17.78 (2.45)
R_{sym} (%)	7.6 (75.1)	4.0 (50.2)
$CC_{1/2}$	99.9 (89.9)	99.9 (82.6)
Refinement		
R_{work}	0.1707	0.1867
R_{free}	0.2013	0.2096
Number of atoms		
Protein	2036	5051
Ligand / Ions	0 / 4	20 / 5
Water	113	329
R.m.s. deviations		
Bond lengths (Å)	0.010	0.009
Bond angles (°)	1.30	1.31

Statistics for the highest-resolution shell are shown in parentheses.

Table S3. Summary of the primers used in the study. The sequences of the primers (ThermoFisher Scientific), in the directions "forward" and then "reverse", are presented from 5 'to 3'. The nucleotides corresponding to the mutated amino acids are shown in red.

**Constructions**	**Sequence (5'-3')**
*Vp*16 PDF(VTI)helices	CTGAACGGCGTAACGATAATGGATTATCTGAGTCCGCTG<br>CAGCGGACTCAGATAATCCATTATCGTTACGCCGTTCAG
*Vp*16 PDF(VTF)helices	CTGAACGGCGTAACGTTTATGGATTATCTGAGTCCGCTG<br>CAGCGGACTCAGATAATCCATAAACGTTACGCCGTTCAG
E. coli<br><br>PDF(KLF)Δhelices	CCTGGTCGGCAAACTGTTTTAGGATTATCTGTCACCGCTG<br>CAGCGGTGACAGATAATCCTAAAACAGTTTGCCGACCAGG
E. coli<br><br>PDF(VTI)helices	CACCTGGTCGGCGTCACCATC ATGGATTATCTGTCACC<br>CAGCGGTGACAGATAATCCTAGATGGTGACGCCGACCAGG
E. coli<br><br>PDF(VTI)Δhelices	CAGCATGAGATGGATCACCTGGTCGGCGTCACCATCTAGGATTATCTG<br>TCACCGCTGAAACAAC<br>GTTGTTTCAGCGGTGACAGATAATCCTAGATGGTGACGCCGACCAGG<br>TGATCCATCTCATGCTG
E. coli<br><br>PDF(KLI)helices	CACCTGGTCGGCAAACTGATTATGGATTATCTGTCACC<br>GGTGACAGATAATCCATAATCAGTTTGCCGACCAGGTG

Table S4. Protein sequence of wild type PDFs and chimeras used in this study. The mutated amino acids with respect to the wild version are shown in red. The protein sequence corresponding to the wild type sequence of *E. coli* PDF is shown in green whereas that of *Vp*16 PDF is shown in black.

PDF	Amino acid sequence
*Vp*16T	MKILKDDAPELHAIAAEVPHGEDVKDLVLDMTAAMTAAGGIGLAGNQVGVLKRIIVLR CPTFKGCVINPIITRHTDGHVYSPEGCLSYPGKTVAKKRRNKVVVEGYDMDWQPITIA AKGLTAFCLQHEIDHLNGVTI
1906	MILQLIPNTHPILHERVKKCSYDLDRAEISKILYENMVHHNGVGLSANQIGINERVFI MVKDLECNEILTCFNPKIVKQSSKTVVMEEGCLSYPDEFIEIERSETVIVKYEDENKV NHKIKLEGFAARVFLHEFDHMQGINFTQR
1577	MILQLIPNTHPLLHERMNKCSYDLDRNEISKILHENMIHHEGVGLSANQIGMSERVFI MMIDTETEETITCFNPRIVKRYDEEVWFEEGCLSYPDEIINIQRPNRIVVKYEDENKV DHKVKLEGFAARVFLHEYDHMEGINFT
2750	MILELTPNTHPILHKKVKPCSYNLDRNFLSKTLIENMIHYNGIGISANQIGIWERAFA MVRDIEHNEIMVCFNPRIIKTYAEEVEMEEGCLSYPKLFLKIKRPDRIVVKYEDVDKK THKVKLQGVASRVFQHEYDHMEGI
*Vp*16 PDF(VTI)helices	MKILKDDAPELHAIAAE*VP*HGEDVKDLVLDMTAAMTAAGGIGLAGNQVGVLKRIIVLR CPTFKGCVINPIITRHTDGHVYSPEGCLSYPGKTVAKKRRNKVVVEGYDMDWQPITIA AKGLTAFCLQHEIDHLNGVTIMDYLSPLKQQRIRQKVEKLDRLKARA
*Vp*16 PDF(VTF)helices	MKILKDDAPELHAIAAE*VP*HGEDVKDLVLDMTAAMTAAGGIGLAGNQVGVLKRIIVLR CPTFKGCVINPIITRHTDGHVYSPEGCLSYPGKTVAKKRRNKVVVEGYDMDWQPITIA AKGLTAFCLQHEIDHLNGVTFMDYLSPLKQQRIRQKVEKLDRLKARA
E. coli PDF(KLF)Δhelices	MAVLQVLHIPDERLRKVAKPVEEVNAEIQRIVDDMFETMYAEEGIGLAATQV DIHQRIIVIDVSENRDERLVLINPELLEKSGETGIEEGCLSIPEQRAL*VP*RA EKVKIRALDRDGKPFELEADGLLAICIQHEMDHLVGKLF
E. coli PDF(VTI)helices	MAVLQVLHIPDERLRKVAKPVEEVNAEIQRIVDDMFETMYAEEGIGLAATQV DIHQRIIVIDVSENRDERLVLINPELLEKSGETGIEEGCLSIPEQRAL*VP*RA EKVKIRALDRDGKPFELEADGLLAICIQHEMDHLVGVTIMDYLSPLKQQRIR QKVEKLDRLKARA
E. coli PDF(VTI)Δhelices	MAVLQVLHIPDERLRKVAKPVEEVNAEIQRIVDDMFETMYAEEGIGLAATQV DIHQRIIVIDVSENRDERLVLINPELLEKSGETGIEEGCLSIPEQRAL*VP*RA EKVKIRALDRDGKPFELEADGLLAICIQHEMDHLVGVTI
E. coli PDF(KLI)helices	MAVLQVLHIPDERLRKVAKPVEEVNAEIQRIVDDMFETMYAEEGIGLAATQV DIHQRIIVIDVSENRDERLVLINPELLEKSGETGIEEGCLSIPEQRAL*VP*RA EKVKIRALDRDGKPFELEADGLLAICIQHEMDHLVGKLIMDYLSPLKQQRIR QKVEKLDRLKARA

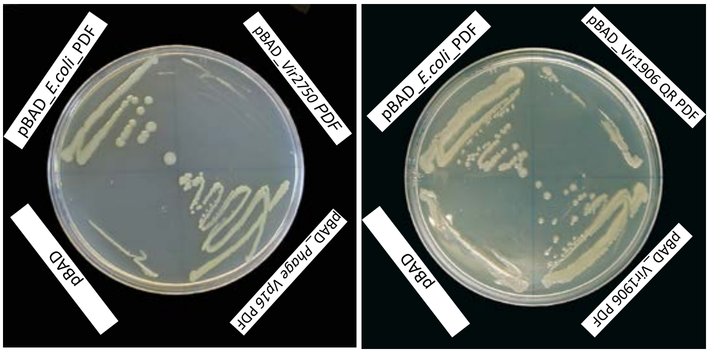
pBAD_E.coli_PDF
pBAD_Vir2750 PDF
pBAD
pBAD_Phage Vp16 PDF
pBAD_E.coli_PDF
pBAD_Vir1906 QR PDF
pBAD
pBAD_Vir1906 PDF

Figure S1

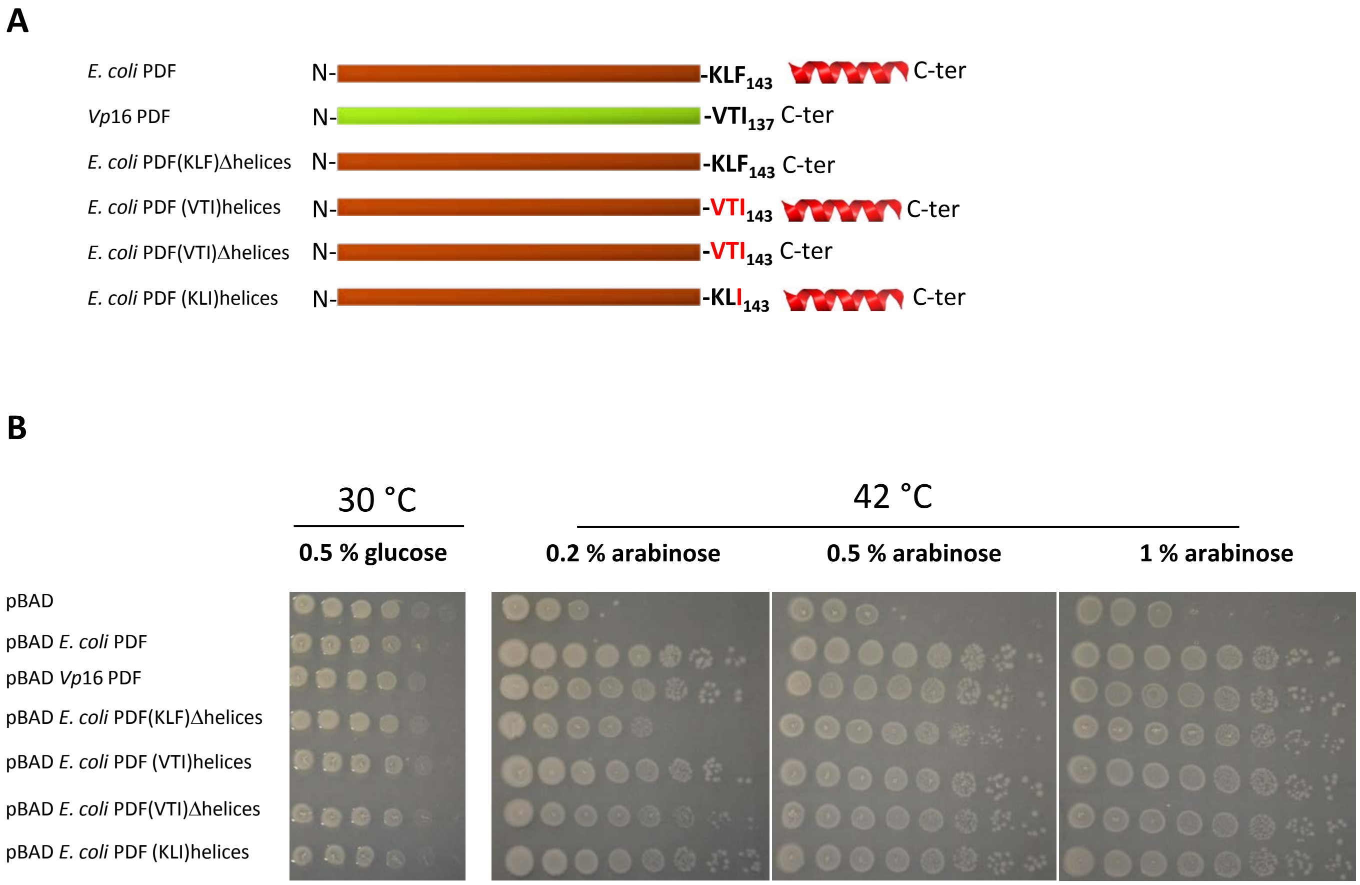
A
E. coli PDF
N-
-KLF143
C-ter
Vp16 PDF
-VTI137
E. coli PDF(KLF)Δhelices
E. coli PDF (VTI)helices
-VTI143
E. coli PDF(VTI)Δhelices
E. coli PDF (KLI)helices
-KLI143
B
30 °C
42 °C
0.5 % glucose
0.2 % arabinose
0.5 % arabinose
1 % arabinose
pBAD
pBAD E. coli PDF
pBAD Vp16 PDF
pBAD E. coli PDF(KLF)Δhelices
pBAD E. coli PDF (VTI)helices
pBAD E. coli PDF(VTI)Δhelices
pBAD E. coli PDF (KLI)helices

Figure S2

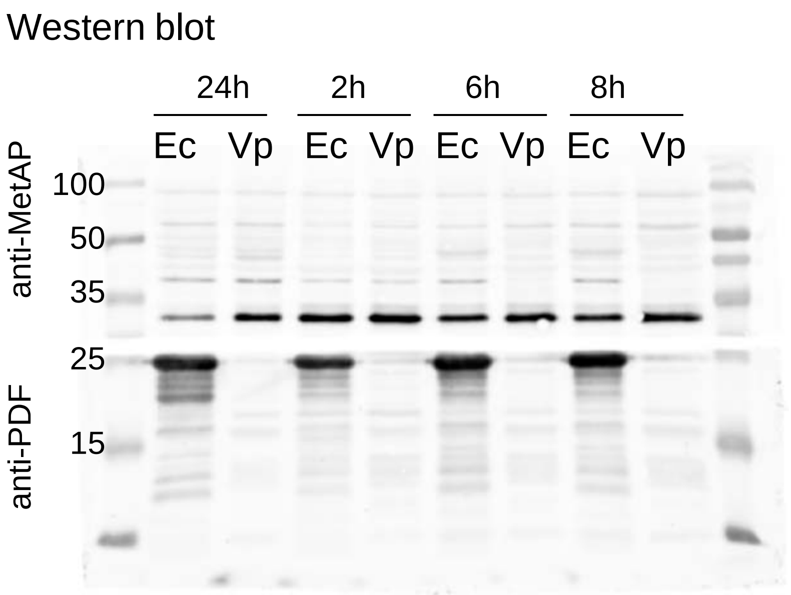
Western blot
24h
2h
6h
8h
Ec Vp Ec Vp Ec Vp Ec Vp
anti-MetAP
anti-PDF
100
50
35
25
15

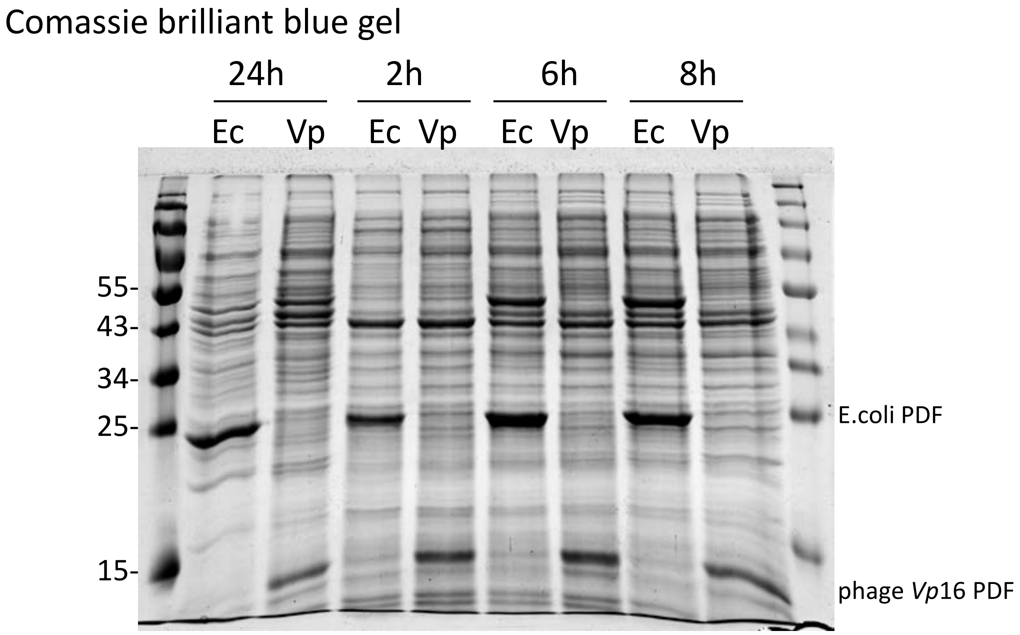
Comassie brilliant blue gel
24h
2h
6h
8h
Ec Vp Ec Vp Ec Vp Ec Vp
55-
43-
34-
25-
15-
E.coli PDF
phage Vp16 PDF

Figure S3
